# Learning the time of pain in the human motor system

**DOI:** 10.1097/j.pain.0000000000003730

**Published:** 2025-08-06

**Authors:** Daniela Dalbagno, Sonia Betti, Sara Garofalo, Vanessa Mannari, Giuseppe di Pellegrino, Francesca Starita

**Affiliations:** a Department of Psychology, University of Bologna, Viale Rasi e Spinelli, Cesena (FC), Italy; b Department of General Psychology, University of Padova, Via Venezia, Padova (PD), Italy

**Keywords:** Pain anticipation, Corticospinal excitability, Event timing; motor cortex, Pavlovian conditioning, Transcranial magnetic stimulation

## Abstract

Although traditionally conceptualized as a sensory phenomenon, pain could also be framed as a motor issue. Yet, the role of the motor system in pain prediction remains poorly understood. Here, we test whether the human motor system can learn to predict the time of pain. In 2 experiments, distinct groups of participants learned that different visual cues predicted pain occurrence either shortly or long after cue onset. Single-pulse transcranial magnetic stimulation was administered over the primary motor cortex to probe corticospinal excitability at 3 critical timepoints during cues presentation, namely long before, immediately before, and long after the time of pain. Bayesian informative hypothesis testing supported, for both experiments, the hypothesis that the motor system learns pain timing through sustained motor inhibition during pain anticipation. Specifically, independently of actual pain occurrence, corticospinal excitability decreased from cue onset, long before the time of pain, until the time of pain, and recovered once this had passed. In addition, stronger corticospinal inhibition long before the time of pain correlated with greater subjective temporal anticipation of pain occurrence, suggesting a role for the motor system in transforming physical time into subjective time. Sustained motor inhibition during pain anticipation may be adaptive for the preparation of prompt defensive behaviors. However, exaggerated motor inhibition may become maladaptive creating an anticipated sense of a pain that may never occur. In clinical settings, enhanced motor inhibition may interfere with therapeutic interventions aimed at restoring motor function and pain resolution.

## Abstract 2

Supplemental Digital Content is Available in the Text.

Regardless of the actual occurrence of pain, corticospinal excitability decreases from long until immediately before the time of pain and recovers once it has passed.

## 1. Introduction

Although traditionally investigated as a sensory problem, pain could be framed as a motor issue. Pain is a powerful motivator of action to minimize current and future harm.^5,42,59,72,73^ Experimental evidence shows that both tonic and phasic painful stimulations exert an inhibitory influence on the motor system by reducing corticospinal excitability^66^ (for a meta-analysis: Ref. 53,65). Moreover, we have recently demonstrated that the mere threat of pain, cued by the presence of the visual stimuli, is sufficient to reduce corticospinal excitability, immediately before (ie, 60 ms) shock occurrence.^8^

**Figure 1.**
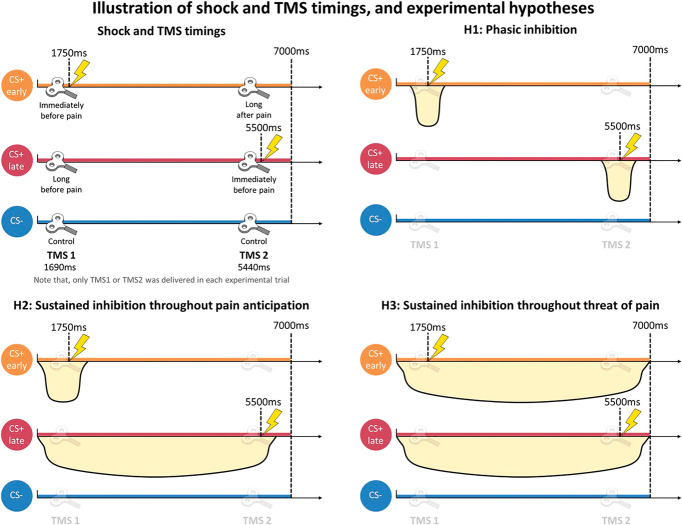
Illustration of shock and TMS timings, and experimental hypotheses. Shock and TMS timings. Timings of electrotactile shocks and TMS pulses used in the Pavlovian threat conditioning task during the presentation of each conditioned stimulus (CS) are represented. Experimental hypotheses. Graphical representation of the 3 experimental hypotheses regarding corticospinal excitability under threat of pain. H1, phasic inhibition hypothesis; H2, sustained inhibition throughout pain anticipation hypothesis; H3, sustained inhibition throughout threat of pain hypothesis. The yellow curved dips indicate when the reduction in corticospinal excitability is expected under each hypothesis.

As organisms learn which stimuli in the environment predict pain, they also learn about the timing of that pain. Indeed, the ability to accurately time defensive responses is crucial for survival.^39,62^ In this regard, the motor cortex is known for its role in event and interval timing, as well as temporal learning, being engaged not only in time perception but also in timed behavior.^16,34,49,52,70^ Considering this evidence, here, we tested whether the inhibition of the motor system under threat of pain^8^ is bound to the time of pain occurrence through 2 experiments. The first aimed at testing our main hypotheses, and the second was designed to exclude any effects of task structure that could have confounded the previous results. In both experiments, we applied single-pulse transcranial magnetic stimulation (TMS) over the primary motor cortex during a Pavlovian threat conditioning task in which we manipulated the temporal imminence of pain.^9,25,56^ Specifically, participants learned that initially neutral visual stimuli predicted pain early, that is, conditioned stimulus early (CS+early), or later, that is, conditioned stimulus late (CS+late), after their appearance. Such manipulation enabled us to test corticospinal excitability in 3 critical timepoints during the threat of pain, marked by CS+ presentation: long before pain, immediately before pain, and long after the time of pain had passed (Fig. 1). Finally, participants completed a time interval reproduction task to assess subjective time estimates and correlate them with corticospinal excitability.

Crucially, we formulated 3 a priori hypotheses (Fig. 1) about the modulations of motor system excitability during the task, inspired by the different brain activity patterns found to be associated with processing time.^78^ The *phasic inhibition hypothesis* (H1) posits that the reduction in corticospinal excitability is observable exclusively immediately before the time of pain occurrence, but not long before or after it. The *sustained inhibition throughout pain anticipation hypothesis* (H2) posits that the reduction in corticospinal excitability starts at threat appearance and is maintained until the time of pain but recovers once the time of pain has passed. The *sustained inhibition throughout threat of pain hypothesis* (H3) posits that the reduction in corticospinal excitability occurs throughout the presentation of the threat, regardless of the actual time of pain.

## 2. Methods

### 2.1. Experiment 1

#### 2.1.1. Participants

A total of 30 healthy adult volunteers participated in the experiment. Two of them were excluded from data analysis because of a difference in baseline amplitude of MEPs recorded before and after the conditioning task (see “Transcranial magnetic stimulation” section below), which could have compromised the integrity of the results of the conditioning task (although additional analyses confirmed that their exclusion did not affect the overall findings). Thus, data from 28 participants (14 female, aged between 19 and 29 years: mean [M] = 25, standard deviation [SD] = 2.973) were included in the data analysis. The sample size was calculated through a power analysis conducted with MorePower 6.0.4^12^ with the following parameters: Analysis: ANOVA; Design factors: repeated measures 3 (CS: CS+early, CS+late, CS−) × 2 (Timing TMS: early, late); Effect of interest: 3 × 2; Alpha: 0.05; Power: 0.8 (partial eta = 0.17, Cohen f = 0.45). The effect size was based on the effect size found in our previous study.^8^ All participants were right-handed, as assessed by the Edinburgh Handedness Inventory,^64^ with normal or corrected-to-normal visual acuity. They were all screened for TMS exclusion criteria to exclude any history of head trauma or head surgery, seizures, and family history of epilepsy, implanted hardware, medications, and neurological and medical illnesses according to safety guidelines.^67,68^ Also, participants had no current neurological, or psychiatric diagnosis or chronic medical condition, as well as no recent history of trauma affecting the upper limbs, nor were they currently suffering from any pain or taking any analgesic medication. The study followed the American Psychological Association Ethical Principles of Psychologists and Code of Conduct and the Declaration of Helsinki and was approved by the Bioethics Committee of the University of Bologna (protocol number 224,364). All participants were naïve to the purposes of the experiment and gave their written informed consent for their participation.

#### 2.1.2. Experimental task

##### 2.1.2.1. Pavlovian threat conditioning task

**Figure 2.**
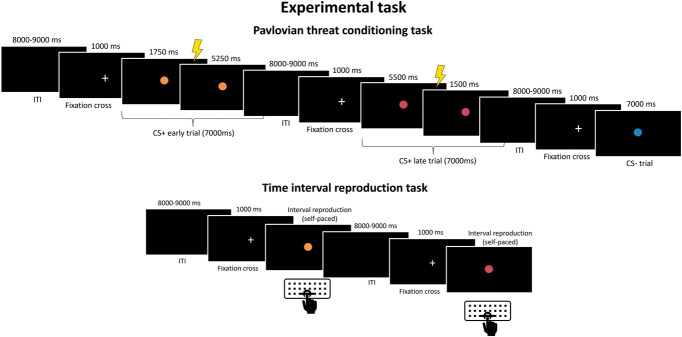
Experimental task. Pavlovian threat conditioning task. The trial structure is represented. Three types of CS, presented for 7000 milliseconds each one, were used: a CS+early associated with shock delivered 1750 milliseconds after stimulus onset (60 ms after TMS 1), a CS+late that was associated with shock delivered 5500 milliseconds after stimulus onset (60 ms after TMS 2), and a CS− never paired with shock. Time interval reproduction task. A CS+ appeared until participants pressed the keyboard space bar, indicating their time estimate of shock occurrence and ending the CS+ reproduction.

Participants completed a Pavlovian threat conditioning task,^51^ during which they learned the association between 3 visual stimuli and their respective outcomes (Fig. 2). The 3 visual stimuli were filled colored circles (64 pixels diameter, blue #5698D4, pink #C760CA, or yellow #F4E634), representing the 3 different conditioned stimuli (CSs). Two of them were CSs+ associated with the delivery of an electrotactile shock to the right forearm, whereas the other was a CS− never associated with shock.

In each experimental trial, one of the CSs appeared in the center of a computer screen on a black background for 7000 milliseconds. Crucially, the 2 CSs+ differed in the timing of shock delivery, specifically, for the CS+early, the shock occurred 1750 milliseconds after CS onset, whereas for the CS+late, the shock occurred 5500 milliseconds after CS onset. The intertrial interval (ITI) was jittered between 8 and 9 seconds, and before the beginning of each trial, a white fixation cross was presented for 1 second in the center of the screen. Each CS was presented 46 times, for a total of 138 trials. For each CS+, the shock occurred in 28 trials (ie, 61% reinforcement rate). Having a partial reinforcement enabled us to verify that the modulations of corticospinal excitability were bound to the time of shock, rather than to its occurrence (or nonoccurrence), as highlighted in the results. The first 3 trials consisted of the presentation of a CS−, a reinforced CS+early, and a reinforced CS+late, in random order, whereas the TMS pulse was never delivered to facilitate the understanding of the CS-shock contingency. The remaining trials proceeded in pseudo-random order with no more than 2 consecutive CSs of the same type in a row. The task was divided into 2 blocks, with a 1-minute break between blocks to reduce participants' fatigue. Color assignment to each CS was counterbalanced among participants.

Before beginning the task, participants were instructed as follows: “You will see a colored circle appearing on the screen. Sometimes, the circle may deliver a shock on your arm. Your task is to understand with which color and when the circle gives you the shock.” Note that no information was provided regarding the shock-time-CS contingency, and participants had to learn this through experience. In addition, they were instructed to observe the screen throughout the task while keeping their hands and arms relaxed, without executing any motor response. During the task, corticospinal excitability was probed during CS presentation by delivering single pulse TMS, as detailed in the transcranial magnetic stimulation section below.

At the end of the task, explicit learning was assessed by having participants rate the valence and arousal of each CS and the CS-shock contingency on a series of 11-point Likert scales (ranging from 0 to 10). The following 3 questions were answered regarding each CS, presented in random order: (1) “When I saw this circle, the feeling I had was” (Valence rating; ranging from 0 = “unpleasant” to 10 = “pleasant”); (2) “Seeing this circle was” (Arousal rating; ranging from 0 = “not activating at all” to 10 = “extremely activating”); (3) “This circle gave me the shock” (Contingency rating; from 0 = “never” to 10 = “always”). This task was preregistered at https://osf.io/z26a4.

##### 2.1.2.2. Time interval reproduction task

To assess learning of the timing of shock delivery for each CS+, participants performed an interval reproduction task, in which they reproduced the time elapsed between the CS+ appearance and shock occurrence (Fig. 2). Thus, in each trial, a CS+ appeared in the center of the screen until the participant pressed the space bar on the computer keyboard, to indicate their subjective estimation of the time of shock occurrence and end CS+ presentation. Spacebar pressing was required only to end the presentation of the CS+, whereas the CS+ appearance was computer-generated (a similar approach to 55). Each CS+ was presented for 5 trials, in random order followed by a jittered 8- to 9-s ITI (steps of 500 ms), then a 1-second fixation cross was presented in the center of the screen before the next CS+ appearance. No shock was delivered during this task.

Given the novelty of the paradigm, and the fact that delivering the TMS pulse during CS presentation prevented the artifact-free recording of psychophysiological measures typical of conditioning paradigms (eg, skin conductance response),^46^ we conducted a preliminary validation experiment to ensure the suitability of the task in inducing a well-established conditioned response in participants and learning of CS-US time intervals. In particular, skin conductance response was assessed as a reliable and validated measure of conditioning,^6,46^ as well as explicit ratings. The results of this validation experiment are reported in the Supplementary Materials (http://links.lww.com/PAIN/C331). Briefly, they support the validity of our paradigm in inducing the acquisition of threat conditioning, showing an increase in skin conductance response (SCR) during the presentation of both CS+early and CS+late, as compared to CS− (see Supplementary materials, http://links.lww.com/PAIN/C331).

#### 2.1.3. Stimulations and recordings for the Pavlovian threat conditioning task

**Figure 3.**
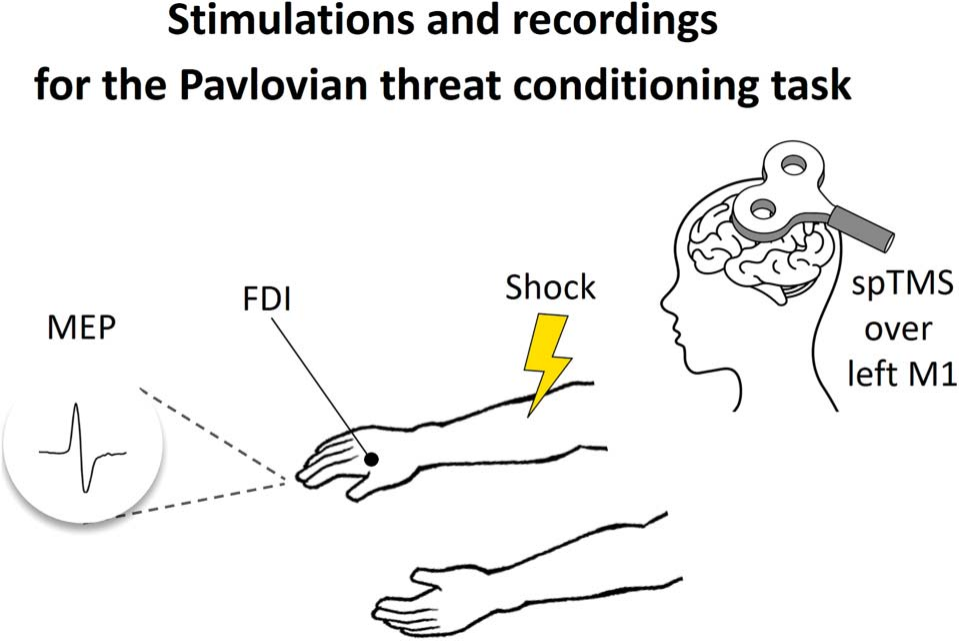
Stimulations and recordings for the Pavlovian threat conditioning task: the shock was delivered at the right extensor carpi radialis muscle, and spTMS was delivered over the left M1 to record MEP from the right FDI of participants.

The experimental setup for the Pavlovian threat conditioning task (Fig. 3) included the delivery of painful electrotactile stimulations (shocks) over the participant's right forearm. In addition, in each trial, TMS pulses were delivered over the left primary motor cortex (M1), whereas electromyography was recorded continuously from the first dorsal interosseus (FDI) muscle of the right hand.

##### 2.1.3.1. Painful electrotactile stimulation

The painful stimulation consisted of a 2-millisecond electrotactile shock generated by a Digitimer Stimulator (Model DS7A, Digitimer Ltd., United Kingdom) and delivered to the participant's right forearm proximal to the extensor carpi radial muscle, through pregelled Ag/AgCl snapped electrodes (Friendship Medical, SEAg-S-15,000/15 × 20). The shock intensity (M = 55.00 mA, SD = 7.07) was calibrated for each participant to a level deemed “at the threshold between highly unpleasant and painful, to the point that if they knew it was coming, they would prefer not to experience it” using an ascending staircase procedure, starting from 0 mA and increasing in steps of 5 mA (as in 72,74–76). When the participant reported that the stimulation intensity level was reached, they rated the shock on a scale ranging from 0 (no sensation) to 10 (untolerable pain), aiming for a rating of 8, both before (M = 7.889, SD = 0.424) and after the conditioning task (M = 7.417, SD = 1.248) to confirm the maintenance of the aversive value of the shock (Bayesian paired sample *t* test BF_10_ = 1.189; err% = 0.022).

##### 2.1.3.2. Transcranial magnetic stimulation

Single-pulse TMS was administered using a 70-mm figure-of-eight coil connected to a Magstim Rapid^2^ stimulator (Magstim Co., Whitland, United Kingdom). Pulses were delivered to the left primary motor cortex (M1) of the participant, in correspondence with the FDI muscle representation. The coil was placed on the head at a 45-degree angle relative to the interhemispheric fissure, with the handle pointing laterally and caudally.^11,54^ Then, to find the FDI muscle representation, the best position for the coil on the scalp at which the lower stimulation intensity elicits the largest MEP, that is, optimal scalp position, was determined by moving the coil in approximately 0.5-cm steps around the presumed hand motor area. The position was then marked on a tight-fitting cap worn by the participants, ensuring a correct coil placement throughout the experiment. For each participant, the intensity of TMS stimulation was set at 120% of the individual resting motor threshold, that is, the lowest stimulation intensity inducing MEPs with at least ≥ 50 µV peak-to-peak amplitude in a relaxed muscle in 5 of 10 trials.^69^ The resting motor threshold ranged from 56% to 83% (mean = 71.25%, SD = 11.148) of the maximum stimulator output.

Corticospinal excitability was probed by delivering single pulse TMS 1690 milliseconds (ie, TMS 1) or 5440 milliseconds (ie, TMS 2) after CS onset (Fig. 1). The timing of the TMS pulse was selected to probe corticospinal excitability at specific moments relative to the time of shock. Specifically, corticospinal excitability was probed immediately before the time of shock (ie, 60 milliseconds before) when TMS 1 was delivered during CS+early trials and when TMS 2 was delivered during CS+late trials. At the same time, these timings also enabled us to probe corticospinal excitability long before (ie, 3810 milliseconds) and long after (ie, 3790 milliseconds) the time of shock, corresponding to TMS 1 in CS+late trials and TMS 2 in CS+early trials, respectively. The same TMS 1 and TMS 2 timings were applied in CS− trials as a control comparison. Also, the timing of TMS 2 ensured that 3750 milliseconds had passed because the shock delivered in CS+early reinforced trials, ruling out the possibility that any observed changes in corticospinal excitability were because of the shock itself. Indeed, previous studies have shown that modulations of corticospinal excitability in response to painful electrotactile^14,27,41,63,79,82^ or laser stimulation^1,19,84,85^ typically last no longer than 2000 milliseconds. Importantly, only a single TMS pulse was delivered per CS trial, to exclude a modulation of corticospinal excitability because of the delivery of repetitive TMS pulses, rather than because of threat conditioning.^13,38^ Finally, to ensure that the occurrence of a TMS pulse could not be predicted during conditioning, for each CS, only 40 out of 46 trials had a TMS pulse. In particular, for each CS+, TMS 1 occurred in 20 trials (12 reinforced and 8 nonreinforced), whereas it did not occur in 3 trials (2 reinforced, 1 nonreinforced); the same happened for TMS 2. To exclude any nonspecific, long-lasting changes in basal corticospinal excitability that could be related to the TMS, the task, or the shocks per se, and thus affecting the results, baseline corticospinal excitability was probed at the beginning and the end of the experimental session. This was done by delivering 15 single TMS pulses and acquiring MEPs while participants passively observed a fixation cross on the screen. An interpulse interval of at least 5000 milliseconds was adopted, thereby avoiding changes in corticospinal excitability because of repeated TMS pulses.^13^ No difference emerged when comparing MEPs recorded during the initial (M = 1.244, 95% credible interval (CI) [0.942-1.576]) and final (M = 1.383, 95% CI [1.035-1.705]) baseline phases (BF_10_ = 0.594; err% = 0.027).

##### 2.1.3.3. Electromyography recording

Surface electromyography (EMG) activity was recorded from the FDI muscle of the participant's right hand at 5000 Hz, with a 5-kHz lowpass filter, through a pair of Ag/AgCl electrodes (BIOPAC EL501) placed in a belly‐tendon montage connected to an EMG100C module of the BIOPAC MP-150 System (Goleta, CA). After skin preparation, a small amount of isotonic hyposaturated conductant gel (Lectron III Gel, NEUROSPEC) was added to the electrodes, which were placed and fixed on the target positions. The active electrode was placed over the muscle belly, determined by palpation during maximum voluntary contraction, whereas the reference and ground electrodes were placed over the proximal interphalangeal juncture and the radial styloid process.

#### 2.1.4. Procedure

Participants were tested individually in a single experimental session lasting approximately 2 hours. They were comfortably seated in a silent room in front of a computer screen (size: 43 inches; resolution: 1920 × 1080 pixels; refresh rate: 60 Hz), at ∼80 cm viewing distance. Then, before Pavlovian threat conditioning, the shock intensity was calibrated. Then, baseline corticospinal excitability was measured. The Pavlovian threat conditioning task followed, after which explicit learning was assessed and the time interval reproduction task performed. Finally, baseline corticospinal excitability was measured again. Participants were debriefed about the experimental hypotheses at the end of the whole experiment. A PC running the OpenSesame software^51^ connected through a NI USB-6281 device (National Instruments, Austin, TX) to a BIOPAC MP-150 System (Goleta, CA), a Digitimer Stimulator (Model DS7A, Digitimer Ltd., United Kingdom), and Magstim Rapid^2^ stimulator (Magstim Co., Whitland, United Kingdom) controlled the flow of the experimental tasks, stimulations delivery, and data recording.

#### 2.1.5. Dependent variables

##### 2.1.5.1. Threat conditioned corticospinal response

Electromyography data were preprocessed offline in MATLAB using custom-made scripts. First, epochs from the time of the TMS pulse to 60 milliseconds after it were extracted from the continuous data for MEP identification and analysis, and epochs from −100 milliseconds to the time of the TMS pulse were extracted to identify any muscular preactivation, before TMS delivery. For each epoch, the min and max MATLAB functions were then used to determine the minimum and maximum peak in the EMG signal. The signal was then visually inspected to ensure the correctness of the automatic procedure. Muscular preactivations were defined as trials in which peaks of EMG activity in the 100-millisecond window preceding the TMS pulse exceeded 2 SD from the mean background EMG activity. MEPs preceded by such preactivation were discarded to prevent contamination of the MEP measurements (a total of 5.57% of MEPs were excluded). This enabled the exclusion of any influence of participants' movement or movement that may have been evoked by shock delivery to influence MEPs recorded on the next trial. For the remaining trials, individual peak‐to‐peak MEP amplitudes (mV) were calculated and considered as a proxy for corticospinal excitability during threat conditioning. Then, MEPs' whose amplitude exceeded ± 2 SD of the mean amplitude for each experimental condition were excluded as outliers (CS+early: 4.38%; CS+late: 3.66%; CS−: 4.02%). To control for interindividual variability in MEP amplitudes, the raw MEP amplitudes were z‐transformed using the average and standard deviation calculated across all CSs, separately for each participant. Z-scored MEP amplitudes were then averaged for each CS and used for statistical analyses.

##### 2.1.5.2. Time interval reproduced

For each CS+, raw participants' response times for each trial (extracted in ms) were averaged and used for statistical analyses.

##### 2.1.5.3. Explicit acquisition of threat conditioning

Participants' explicit ratings of valence, arousal, and CS-US contingency for each CS were used in statistical analyses to compare responses between CSs.

#### 2.1.6. Statistical analyses

##### 2.1.6.1. Threat conditioned corticospinal response

Given our 3 a priori hypotheses on the corticospinal response during threat of pain (Fig. 1), a Bayesian model comparison approach, namely Bayesian informative hypothesis testing,^28,29,31,36^ was chosen for statistical analysis. This approach enables us to quantify how much more likely the data are under a given hypothesis as compared to the other experimental hypotheses tested.^28,37^ Bayesian informative hypothesis can be seen as an extension of rmANOVA, allowing for specification and testing structured hypotheses about relationships between means, rather than solely assessing overall differences, as done by classically defined alternative hypotheses. Indeed, research hypotheses in psychological and cognitive sciences often extend beyond the classical null and alternative framework. Each model expressed a specific hypothesis that could be defined in terms of equality and/or inequality constraints among the parameters. ^7,29,32,36,37,40^ The prior distribution in our models is assumed to be equal among the hypotheses, this means that we manipulated the alternative hypotheses tested in each model, but not the associated prior distribution. For each hypothesis, the posterior model probability (PMP) was calculated via the Bayes theorem and expressed with a value between 0 and 1. This value can be interpreted as the relative amount of support for each hypothesis given the data and the set of competing hypotheses included, namely, the unconstrained hypothesis (Hu), which is a hypothesis representing all possible sets of relationships between the parameters without constraints, and the complement hypothesis (Hc), which is a model that contains any set of restrictions between the parameters except the one represented by the hypothesis. ^28,37^ The posterior model probability associated with each hypothesis is, by default, calculated in 3 ways, that is relative to all other hypotheses tested (PMPa); all other hypotheses tested plus the unconstrained hypothesis Hu (PMPb); and all other hypotheses tested plus the complement hypothesis Hc (PMPc). The model with the highest PMP reflects the hypothesis that best describes the observed data (ie, the hypothesis with the highest relative probability).^28,35^ The Bayes factor matrix was also calculated to show the Bayes factor deriving from the ratio between the marginal likelihoods of all possible pairs of hypotheses tested.^28,35^ To perform these analyses, the “bain” package^28,30^ was implemented in R software (v4.2.1)^65^ and RStudio (v2022.07.2 Build 576).

Specifically, we formulated 3 a priori hypotheses to describe the dynamics of motor system excitability under threat of pain, which were tested against each other using Bayesian informative hypotheses testing (Fig. 1).

(1) H1: Phasic inhibition hypothesis. Motor inhibition is selectively tuned to the time of pain occurrence. Thus, the reduction in corticospinal excitability is observed exclusively immediately before the time of shock (CS+early TMS 1 and CS+late TMS 2) but not long before (CS+late TMS 1) or after (CS+early TMS 2) it, or during CS− presentation (CS− TMS 1 and CS− TMS 2). This hypothesis can formally be represented as follows:

H1:(CS+late TMS1=CS− TMS1)>CS+early TMS1

&

CS+late TMS1>CS+late TMS2

&

(CS+early TMS2=CS− TMS2)>CS+late TMS2

(2) H2: Sustained inhibition throughout pain anticipation hypothesis. Motor inhibition starts at the appearance of the CS+, persists throughout the time preceding pain occurrence, and then recovers once it has passed. Thus, in this case, the reduction in corticospinal excitability is already observed long before the time of shock (CS+late TMS 1), as well as immediately before it (CS+early TMS 1 and CS+late TMS 2), but not long after it (CS+early TMS 2), or during CS− presentation (CS− TMS 1 and CS− TMS 2). This hypothesis can formally be represented as follows:

H2:(CS+late TMS1=CS+early TMS1)<CS−TMS1

&

CS+late TMS1=CS+late TMS2

&

(CS+early TMS2=CS−TMS2)>CS+late TMS2

(3) H3: Sustained inhibition throughout threat of pain hypothesis. Motor inhibition persists during the entire time of CS+ presentation, regardless of the actual time of pain. The rationale is that as learning progresses, the CS+ acquires the same aversive value as the painful shock outcome. Thus, in this case, the reduction in corticospinal excitability is observed as long as a CS+ is present on the screen regardless of the time of shock occurrence, namely at CS+early TMS 1, CS+early TMS 2, CS+late TMS 2, and CS+late TMS 1, but not during CS− presentation (CS− TMS 1 and CS− TMS 2). This hypothesis can formally be represented as follows:

H3:CS+early TMS1=CS+early TMS2

&

(CS+early TMS1=CS+late TMS1)<CS−TMS1

&

(CS+early TMS2=CS+late TMS2)<CS−TMS2

Finally, we included a fourth hypothesis, representing the null hypothesis, assuming no differences among all conditions.

(4) H0: Null hypothesis. No significant differences exist among the different CS conditions or across different TMS timings.

H0:CS+early TMS1=CS+late TMS1=CS−TMS1

&

CS+early TMS1=CS+early TMS2

&

CS+late TMS1=CS+late TMS2

&

CS−TMS1=CS−TMS2

Then, to confirm that changes in corticospinal excitability were driven by the time of shock, rather than shock occurrence per se, a Bayesian paired sample *t* test was conducted comparing the amplitude of averaged MEPs recorded long after the time of pain (TMS 2) for reinforced and nonreinforced CS+early trials. The Bayes Factor (BF_10_) is reported as the probability of the data under the alternative hypothesis (H_1_) over the null hypothesis (H_0_), along with its estimated proportional error (err%).^2,43^

##### 2.1.6.2. Time interval reproduced

To test differences in the time interval reproduced for each CS+, Bayesian paired sample *t* test and Bayesian analyses of variance (ANOVA) were, respectively, used. The Bayes Factor (BF_10_) is reported as the probability of the data under the alternative hypothesis (H_1_) over the null hypothesis (H_0_), along with its estimated proportional error (err%).^2,43^

##### 2.1.6.3. Explicit acquisition of threat conditioning

Bayesian repeated measures analyses of variance (rmANOVA) and post-hoc comparisons were performed to test differences in valence, arousal, and shock contingency ratings between CSs. The Bayes Factor (BF_10_) is reported as the probability of the data under the alternative hypothesis (H_1_) over the null hypothesis (H_0_), along with its estimated proportional error (err%).^2,43^

All data, analysis code, and research materials will be made available to other researchers in a timely manner upon request.

### 2.2. Experiment 2

Given the results of experiment 1, we questioned whether the inhibition observed long before the time of pain for the CS+late (ie, at TMS 1) indeed represented the dynamic of the motor system's response under threat of pain, or if this inhibition was driven by the structure of the threat conditioning task, where both CS+early and CS+late were presented in the same experimental block. Specifically, we considered whether the shock delivered 1750 milliseconds after CS+early onset may have established a temporal association that generalized to the CS+late. If this were the case, the observed reduction in corticospinal excitability long before the time of pain during the CS+late presentation should be interpreted as a threat-conditioned response generalization^15,20,21,74^ because of the presence of CS+early, rather than as a sustained inhibition of the motor system under threat of pain. Thus, experiment 2 was conducted to disambiguate between these 2 interpretations of the results.

A new group of participants completed a Pavlovian threat conditioning task. This included the same experimental conditions as experiment 1, with the key difference that CS+early and CS+late were conditioned in separate blocks. Specifically, one-half of the participants experienced first a threat conditioning block with CS+early and CS−, and then a conditioning block with CS+late and a different CS−. The other half had the order of the conditioning blocks reversed. The time interval reproduction task was also completed, as in experiment 1, but at the end of each conditioning block.

#### 2.2.1. Participants

Twenty-eight healthy right-handed volunteers (16 women, aged between 18 and 30 years: M = 22, SD = 1.71 years) with the same characteristics as those participating in experiment 1 were tested. No participant who took part in experiment 1 was recruited for experiment 2, to prevent any effect of having previously participated in a similar threat conditioning experiment from influencing the results of experiment 2.

#### 2.2.2. Experimental tasks

##### 2.2.2.1. Pavlovian threat conditioning task

We modified the conditioning task described in experiment 1 such that CS+early and CS+late were conditioned in 2 separate blocks, which we will refer to as Block early and Block late, respectively. Each block included a different CS+ (early or late) and a different CS−, as control stimulus. Thus, the conditioning task included a total of 4 different CSs, which were circles (64 pixels diameter) colored blue (#5698D4), pink (#C760CA), green (#51D968), or yellow (#F4E634). Importantly, within each block, the trial structure and timings, number of trials for each CS, and types and number of TMS pulses for each CS were the same as for experiment 1. The order of the blocks and the color assigned to each CS were counterbalanced among participants.

##### 2.2.2.2. Time interval reproduction task

At the end of each block of the threat conditioning task, participants performed the interval reproduction task, structured as in experiment 1, with the exception that it included only the CS+ presented in that block.

#### 2.2.3. Stimulation and recordings

We used the same parameters of experiment 1 to set up the experimental stimulations and recordings.

##### 2.2.3.1. Painful electrotactile stimulation

The shock intensity (M = 75.54 mA, SD = 22.34) was calibrated for each participant as in experiment 1. Participants rated the unpleasantness of the shock on a scale ranging from 0 to 10, from “no sensation” to “untolerable pain,” both before (M = 7.46, SD = 1.32) and at the end of the experiment (M = 7.00 SD = 1.49) to confirm the maintenance of the aversive value of the shock (Bayesian paired sample *t* test BF_10_ = 1.993; err% = 1.439e-6).

##### 2.2.3.2. Transcranial magnetic stimulation

The resting motor threshold ranged from 57% to 83% (M = 71.18%, SD = 8.99) of the maximum stimulator output.

##### 2.2.3.3. Electromyography recording

As in experiment 1, trials in which peaks of EMG activity in the 100 milliseconds window preceding the TMS pulse exceeded 2 SD from the mean background EMG activity were discarded to prevent contamination of the MEP measurements (a total of 6.54% of MEPs were excluded). In addition, values that exceeded ± 2 SD of the mean amplitude for each CS condition were excluded as outliers (in Block early CS+early: 3.57%, CS−: 3.39%; in Block late CS+late: 4.02%, CS−: 3.84%). MEP data recorded from one participant were excluded because of technical issues during the registration.

As in experiment 1, no differences emerged when comparing MEPs recorded during the initial (M = 0.958, 95% (CI) [0.730-1.254]) and final (M = 0.951, 95% (CI) [0.716-1.159]) baseline phases (BF_10_ = 0.27; err% = 0.031). This indicates that TMS, the task, or the shocks per se did not induce any generalized, long-lasting changes in motor excitability during the experiment.

#### 2.2.4. Procedure

The experimental procedure followed was the same as described in experiment 1.

#### 2.2.5. Dependent variables

Data regarding threat conditioned corticospinal response, explicit acquisition of threat conditioning, and time interval reproduced were recorded and preprocessed as in experiment 1.

#### 2.2.6. Statistical analysis

##### 2.2.6.1. Threat conditioned corticospinal response

Bayesian informative hypotheses were used to test if the pattern of results observed in experiment 1 would be replicated also in experiment 2. More specifically, we conceptually retained the same 3 hypotheses of experiment 1 but adapted them to the experimental conditions of experiment 2. In this case, CS-early refers to the CS− presented in the block together with the CS+early, and CS-late refers to the CS− presented in the block together with the CS+late. We then proceeded to test the 3 hypotheses against each other as previously described for experiment 1.

(1) H1: Phasic inhibition hypothesis. Motor inhibition is selectively tuned to the time of pain occurrence. Thus, the reduction in corticospinal excitability is observed exclusively immediately before the time of shock (CS+early TMS 1 and CS+late TMS 2) but not long after (CS+early TMS 2) or before (CS+late TMS 1) it, or during CS− presentation (CS−early TMS 1, CS−early TMS 2, CS−late TMS 1, and CS−late TMS 2). This hypothesis can formally be represented as follows:

H1:CS+early TMS1<CS−early TMS1

&

CS+early TMS2=CS−early TMS2

&

CS+late TMS1=CS−late TMS2

&

$$\text{CS}+{\text{late}}_{\text{TMS}1}>\text{CS}+\text{late }\text{TMS}2$$

&

CS+late TMS2<CS−late TMS2

(2) H2: Sustained inhibition throughout pain anticipation hypothesis. Motor inhibition persists throughout the time preceding pain occurrence and recovers once this has passed. Thus, the reduction in corticospinal excitability is already observed long before the time of shock (CS+late TMS 1), as well as immediately before it (CS+early TMS 1 and CS+late TMS 2), but not long after it (CS+early TMS 2), or during CS− presentation (CS−early TMS 1 and CS−early TMS 2, CS−late TMS 1, and CS−late TMS 2). This hypothesis can formally be represented as follows

H2:CS+early TMS1<CS−early TMS1

&

CS+early TMS2=CS−early TMS2

&

(CS+late TMS1=CS−late TMS2)<(CS+late TMS1=CS−late TMS2)

(3) H3: Sustained inhibition throughout threat of pain hypothesis. Motor inhibition persists during the entire time of CS+ presentation, regardless of the actual timing of pain. Thus, in this case, the reduction in corticospinal excitability is observed as long as a CS+ is presented on the screen, namely at CS+early TMS 1, CS+early TMS 2, CS+late TMS 1, and CS+late TMS 2, but not during CS− presentation (CS−early TMS 1, CS−early TMS 2, CS−late TMS 1, and CS−late TMS 2). This hypothesis can formally be represented as follows:

H3:(CS+early TMS1=CS+early TMS2)<(CS−early TMS1=CS−early TMS2)

&

(CS+late TMS1=CS+late TMS2)<(CS−late TMS1=CS−late TMS2)

Finally, we included a fourth hypothesis, representing the null hypothesis, assuming no differences amongst all conditions.

(4) H0: Null hypothesis. No significant differences exist among the different CS conditions or across different TMS timings.

H0:CS+earlyTMS1=CS+earlyTMS2=CS−early TMS1=CS−early TMS2

&

CS+lateTMS1=CS+late TMS2=CS−late TMS1=CS−late TMS2

Then, to confirm that changes in corticospinal excitability were driven by the time of shock, rather than shock occurrence per se, a Bayesian paired sample *t* test was conducted comparing the amplitude of averaged MEPs recorded long after the time of pain (TMS 2) for reinforced and nonreinforced CS+early trials.

##### 2.2.6.2. Time interval reproduced

The statistical analysis conducted on reproduced time intervals was performed using the same Bayesian inferential approach as in experiment 1.

##### 2.2.6.3. Explicit acquisition of threat conditioning

Bayesian paired sample t-tests were performed to test differences in valence, arousal, and shock contingency ratings between CSs, separately for the Block CS+early and Block CS+late.

## 3. Results

### 3.1. Experiment 1

#### 3.1.1. Threat conditioned corticospinal response

Posterior model probabilities were used to test our 3 main experimental hypotheses. They showed that H2 was associated with the highest probability, relative to the other experimental or null hypotheses (Table 1; Fig. 7); thus, indicating this as the strongest hypothesis both when excluding (PMPa) or including (PMPb) the unconstrained hypothesis (Hu) and when including the complementary hypothesis (Hc, see PMPc). In line with this, H2 also presented the highest Bayes Factor relative to Hc and Hu (see BFc and BFu in Table 1, respectively). In addition, as reported in the Bayes factor matrix in Table 2, the direct comparison between how likely the data are under each pair of informative hypotheses showed that the support in the data is larger for H2 than for H1, H3, and H0 (BF_21_ = 7.998, BF_23_ = 5.709, and H_20_ = 1383.171). Overall, these results offer convergent support for H2, that is, the sustained inhibition throughout pain anticipation hypothesis, which indicates a reduction in corticospinal excitability that starts long before the time of shock, is maintained also immediately before the time of shock, and finally recovers once the time of shock has passed.

**Table 1:** Model comparison via Bayesian informative hypothesis testing.

	Fit	Com	BFu	BFc	PMPa	PMPb	PMPc
H1	0.177	0.100	1.763	1.927	0.096	0.091	0.092
H2	3.772	0.267	14.105	14.105	0.769	0.729	0.732
H3	0.700	0.283	2.470	2.470	0.135	0.128	0.128
H0	0.921	90.328	0.010	0.010	0.001	0.001	0.001
Hu						0.052	
Hc	0.823	0.900	0.915				0.047

Fit, data fitting index; Com, complexity of the hypotheses; BFu, Bayes Factors of the hypothesis in the row vs the unconstrained hypothesis (Hu) and complement hypothesis (Hc); BFc, Bayes Factors of the hypothesis in the row vs the complement hypothesis (Hc); PMPa, posterior model probability excluding the unconstrained hypothesis; PMPb, posterior model probability including the unconstrained hypothesis; PMPc, posterior model probability including the complement hypothesis.

**Table 2:** Bayes factor matrix.

	H1	H2	H3	H0
H1	1	0.125	0.714	172.933
H2	7.998	1	5.709	1383.171
H3	1.401	0.175	1	242.264
H0	0.006	0.001	0.004	1

Bayes Factors deriving from the ratio between the marginal likelihoods of all possible pairs of hypotheses tested are represented.

To confirm that the recovery of corticospinal excitability is bound to the time of shock, rather than to the delivery of the shock, we conducted a Bayesian paired sample *t* test on MEPs recorded at TMS 2 for the CS+early for shocked vs nonshocked trials. Indeed, no differences emerged between shocked (M = 0.040, 95% credible interval (CI) [−0.095 to 0.183]), and nonshocked CS+early trials (M = 0.059, 95% (CI) [−0.114 to 0.282]; BF_10_ = 0.215; err% = 0.031), confirming that the recovery in corticospinal excitability after the time of shock for CS+early was driven shock timing, rather than shock occurrence.

**Figure 4.**
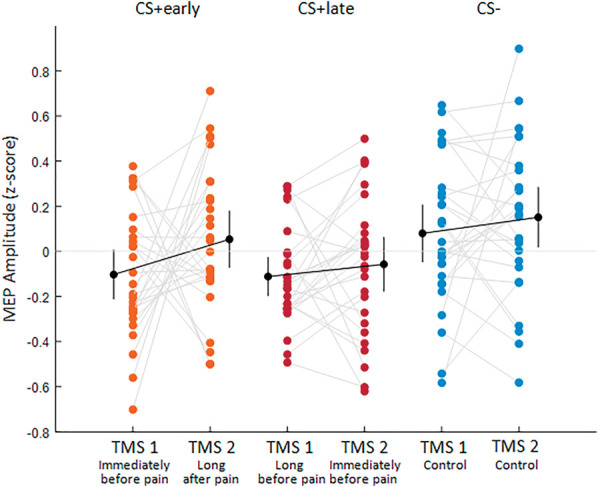
The plot shows individual participants' data (colored dots), group means (black dots), and 95% credible intervals (vertical black lines) of MEP amplitudes (z-scored) for CS+early (orange), CS+late (red), and CS− (blue), as a function of the time of TMS delivery, that is, TMS 1 or TMS 2. Each paired set of observations is connected by a gray line.

Figure 4 shows a descriptive representation of the data, illustrating that, relative to the CS− (TMS 1: M = 0.079, 95% CI [−0.047 to 0.206]; TMS 2: M = 0.153, 95% CI [0.018-0.284]) a reduction in corticospinal excitability can be observed in presence of the CS+early immediately before (M=−0.108, 95% CI [−0.212 to 0.006]) but not long after (M = 0.048, 95% CI [−0.072 to 0.179]) the time of pain, whereas with the CS+late, this occurs both long (M = −0.109, 95% CI [−0.197 to −0.027]) and immediately (M = −0.063, 95% CI [−0.177 to 0.062]) before the time of pain.

#### 3.1.2. Time interval reproduced

**Figure 5.**
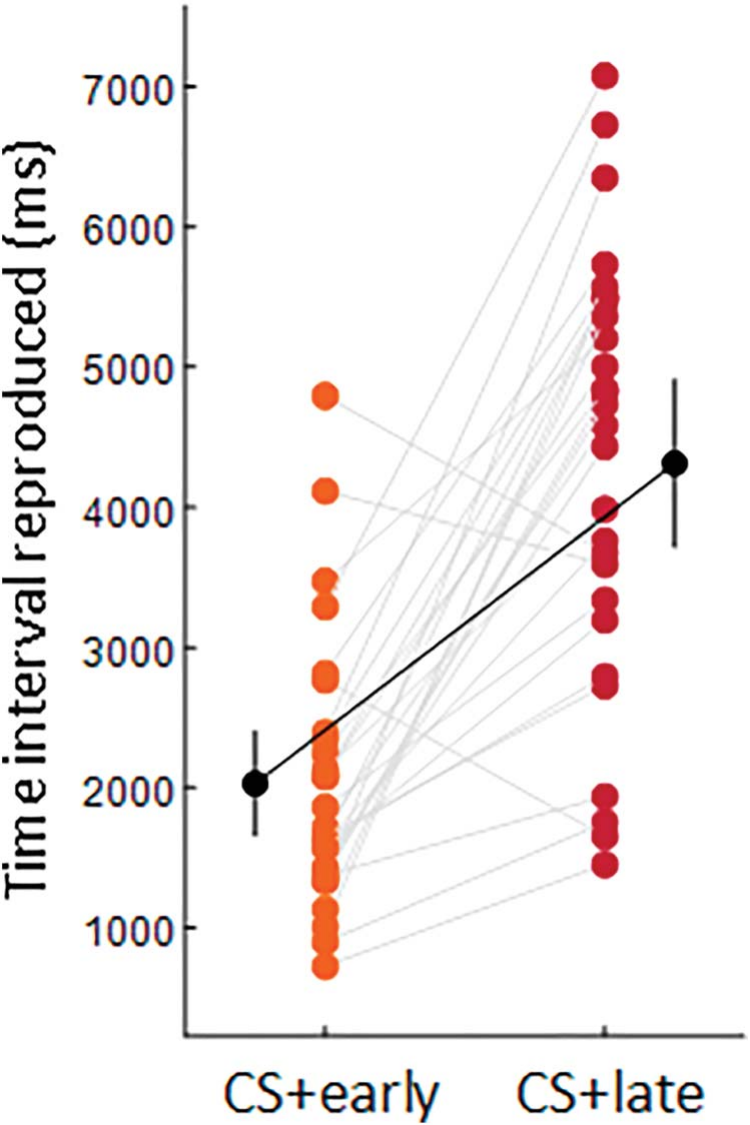
The plot shows individual participants' data (colored dots), group means (black dots), and 95% credible intervals (vertical black lines) of the time interval reproduced (ms) for CS+early (orange) and CS+late (red). Each paired set of observations is connected by a gray line.

A Bayesian paired sample *t* test comparing the mean time intervals reproduced for the CS+early and CS+late showed that participants reproduced a shorter interval for CS+early than CS+late (CS+early: M = 2029.150, 95% CI [1655.234-2403.066], CS+late: M = 4314.221, 95% CI [3715.333-4913.110]; BF_10_ = 193,448.875, err% = 2.730e-10), meaning that participants learned to discriminate the time of shock between the 2 CSs+ (Fig. 5). In addition, a Bayesian paired sample *t* test comparing the difference between the mean reproduced time interval and the actual time of the shock for CS+early (1750 ms—mean reproduced time for CS+early) and CS+late (5500 ms—mean reproduced time for CS+late) revealed that this difference was smaller for CS+early than for CS+late (CS+early: M = −279.150, 95% CI [−653.066 to 94.766]; CS+late: M = 1185.779, 95% CI [586.890-1784.667]; BF_10_ = 349.609, err% = 2.046e-8), indicating that participants were more accurate in reproducing the time interval for the shock early. Finally, a Bayesian paired sample *t* test comparing the standard deviation of time intervals reproduced for the CS+early and CS+late showed lower variability for the CS+early than CS+late (CS+early: M = 354.073, 95% CI [265.787-442.358], CS+late: M = 495.835, 95% CI [397.040-594.630]; BF_10_ = 10.814, err% = 4.992e-8), meaning that participants exhibited greater variability in reproducing the time interval for the shock late. In addition, a Bayesian paired sample *t* test comparing the difference between the actual time of shock and the mean reproduced time interval for CS+early (1750 ms—mean reproduced time for CS+early) and CS+late (5500 ms—mean reproduced time for CS+late) revealed that this difference was smaller for CS+early than for CS+late (CS+early: M = −279.150, 95% CI [−653.066 to 94.766]; CS+late: M = 1185.779, 95% CI [586.890-1784.667]; BF_10_ = 349.609, err% = 2.046e-8), indicating that participants were more accurate in reproducing the time interval for the early shock. Finally, a Bayesian paired sample *t* test comparing the standard deviation of time intervals reproduced for the CS+early and CS+late showed lower variability for the CS+early than CS+late (CS+early: M = 354.073, 95% CI [265.787-442.358], CS+late: M = 495.835, 95% CI [397.040-594.630]; BF_10_ = 10.814, err% = 4.992e-8), meaning that participants exhibited greater variability in reproducing the time interval for the late than the early shock.

#### 3.1.3. Explicit acquisition of threat conditioning

Three Bayesian rmANOVA and post-hoc comparisons were performed with CS (CS+early, CS+late, CS−) as a within‐subject factor to analyze CSs valence, CSs arousal, and shock contingency ratings. A main effect of CS emerged for valence (BF_10_ = 8.683e + 18, err% = 2.522), arousal (BF_10_ = 3.216e + 30, err% = 3.310), and contingency (BF_10_ = 6.815e + 40, err% = 1.507). Participants rated the CS+early (M = 2.929, 95% CI [2.294-3.135]) and the CS+late (M = 2.714, 95% CI [1.758-3.1]) as less pleasant than the CS− (M = 6.536, 95% CI [6.356-7.787]; BF_10_ = 3.043e + 9; err% = 1.821e-12; BF_10_ = 2.136e + 8; err% = 7.017e-12, respectively), whereas ratings for CS+early and CS+late did not differ (BF_10_ = 0.326, err% = 0.03). Moreover, participants rated the CS+early (M = 7.429, 95% CI [7.028-7.829]) and the CS+late (M = 8.071, 95% CI [7.524-8.619]) as more arousing compared to the CS− (M = 1.143, 95% CI [0.486-1.8]; BF_10_ = 2.010e + 12, err% = 3.106e-14; BF_10_ = 7.388e + 11, err% = 7.722e-14, respectively), whereas ratings for CS+early and CS+late did not differ relevantly (BF_10_ = 1.121, err% = 0.023). Regarding the CS-US contingency rating, participants attributed greater contingency to the CS+early (M = 7.464, 95% CI [7.023-7.906]) and CS+late (M = 7.214, 95% CI [6.829-7.6]) than the CS− (M = 0.357, 95% CI [−0.055 to 0.769]; BF_10_ = 4.888e + 15, err% = 4.419e-19; BF_10_ = 3.406e + 15, err% = 4.726e-19, respectively), whereas ratings for CS+early and CS+late did not differ (BF_10_ = 0.356, err% = 0.030).

### 3.2. Experiment 2

#### 3.2.1. Threat conditioned corticospinal response

Similarly, to experiment 1, posterior model probabilities were used to test our 3 main experimental hypotheses. They showed that H2 was associated with the highest probability, relative to the other experimental or null hypotheses (Table 3; Fig. 7); thus, indicating this as the strongest hypothesis both when excluding (PMPa) or including (PMPb) the unconstrained hypothesis (Hu) and when including the complementary hypothesis (Hc, see PMPc). In line with this, H2 also presented the highest Bayes Factor relative to Hc and Hu (see BFc and BFu in Table 3, respectively). In addition, as reported in the Bayes factor matrix in Table 4, the direct comparison between how likely the data are under each pair of informative hypotheses showed that the support in the data is larger for H2 than for H1, H3, and H0 (BF_21_ = 25.199, BF_23_ = 1177.134, and BF_20_ = 1.561e+13). Overall, these results confirm the support for H2, that is, the sustained inhibition throughout pain anticipation hypothesis, which indicates a reduction in corticospinal excitability that starts long before the time of shock, is then found also immediately before the time of shock, and finally recovers once the time of shock has passed, excluding carryover or generalization effect of the timing of pain for CS+early onto the CS+late.

**Table 3:** Model comparison via Bayesian informative hypothesis testing.

	Fit	Com	BF.u	BF.c	PMPa	PMPb	PMPc
H1	0.547	1.15	0.476	0.476	0.031	0.029	0.029
H2	3.095	0.239	12.955	12.955	0.846	0.794	0.795
H3	0.00	0.027	0.012	0.012	0.001	0.001	0.001
H0	0.000	363.691	0.000	0.000	0.000	0.000	0.000
Hu						0.064	
Hc	1.000	0.973	1.028				0.066

Fit, data fitting index; Com, complexity of the hypotheses; BFu, Bayes Factors of the hypothesis in the row vs the unconstrained hypothesis (Hu) and complement hypothesis (Hc); BFc, Bayes Factors of the hypothesis in the row vs the complement hypothesis (Hc); PMPa, posterior model probability excluding the unconstrained hypothesis; PMPb, posterior model probability including the unconstrained hypothesis; PMPc, posterior model probability including the complement hypothesis.

**Table 4:** Bayes factor matrix.

	H1	H2	H3	H0
H1	1	0.040	46.713	6.196e+11
H2	25.199	1	1177.134	1.561e+13
H3	0.021	0.001	1	1.326e+10
H0	0.000	0.000	0.000	1

Bayes Factors deriving from the ratio between the marginal likelihoods of all possible pairs of hypotheses tested are represented.

Then, the Bayesian paired sample *t* test on MEPs recorded at TMS 2 for the CS+early for shocked vs nonshocked trials confirmed, as in experiment 1, that the changes in corticospinal excitability were driven by the time of shock, rather than shock occurrence per se, as no differences emerged between shocked (M = 0.228, 95% CI [0.048-0.444]) and nonshocked trials (M = 0.121, 95% CI [−0.017 to 0.270]; BF_10_ = 0.343; err% = 0.030).

**Figure 6.**
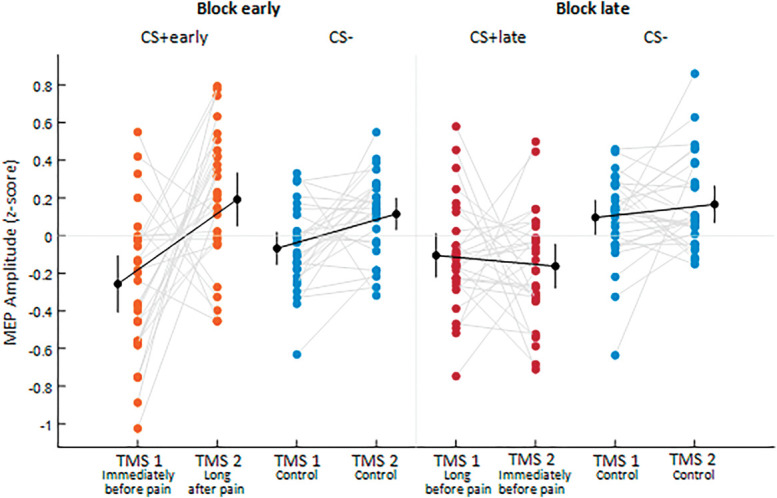
The plot shows individual participants' data (colored dots), group means (black dots), and 95% confidence intervals (vertical black lines) of MEP amplitudes (z-scored) for CS+early (orange), CS+late (red), and CS− (blue), as a function of TMS delivery, that is, TMS 1 or TMS 2, in Block early and Block late. Each paired set of observations is connected by a gray line.

In Figure 6, a descriptive representation of the results is shown. In the Block early, we found a corticospinal excitability reduction in the presence of the CS+early immediately before (M = −0.229, 95% CI [−0.407 to −0.107]) but not long after (M = 0.184, 95% CI [0.051-0.333]) pain, nor in the presence of the CS− in both the control TMS stimulation timepoints (TMS 1: M = −0.068, 95% CI [−0.154 to 0.018]; TMS 2: M = 0.108, 95% CI [0.031-0.199]). In the Block late, we found a corticospinal excitability reduction in the presence of the CS+late both long (TMS1: M = −0.094, 95% CI [−0.221 to 0.011]) and immediately (TMS2: M = −0.176, 95% CI [−0.279 to −0.046]) before pain, whereas corticospinal excitability was not reduced in the presence of the CS− in both the control TMS stimulation timepoints (TMS 1: M = 0.094, 95% CI [−0.006 to 0.187]; TMS 2: M = 0.170, 95% CI [0.069-0.263]).

**Figure 7.**
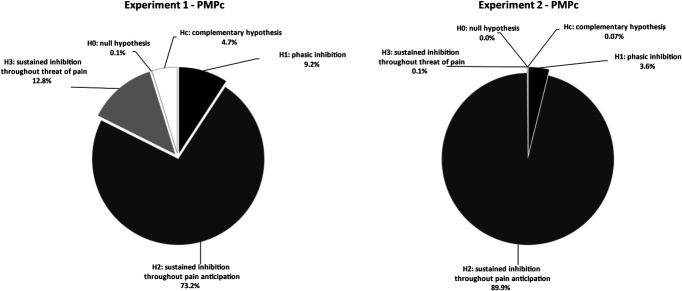
Bayesian informative hypothesis testing. The pie charts represent the posterior model probabilities associated with the 4 models (H1, H2, H3, and H0) when including the complementary (Hc) hypothesis. Results of experiment 1 (left panel) and experiment 2 (right panel) are represented.

#### 3.2.2. Time interval reproduced

**Figure 8.**
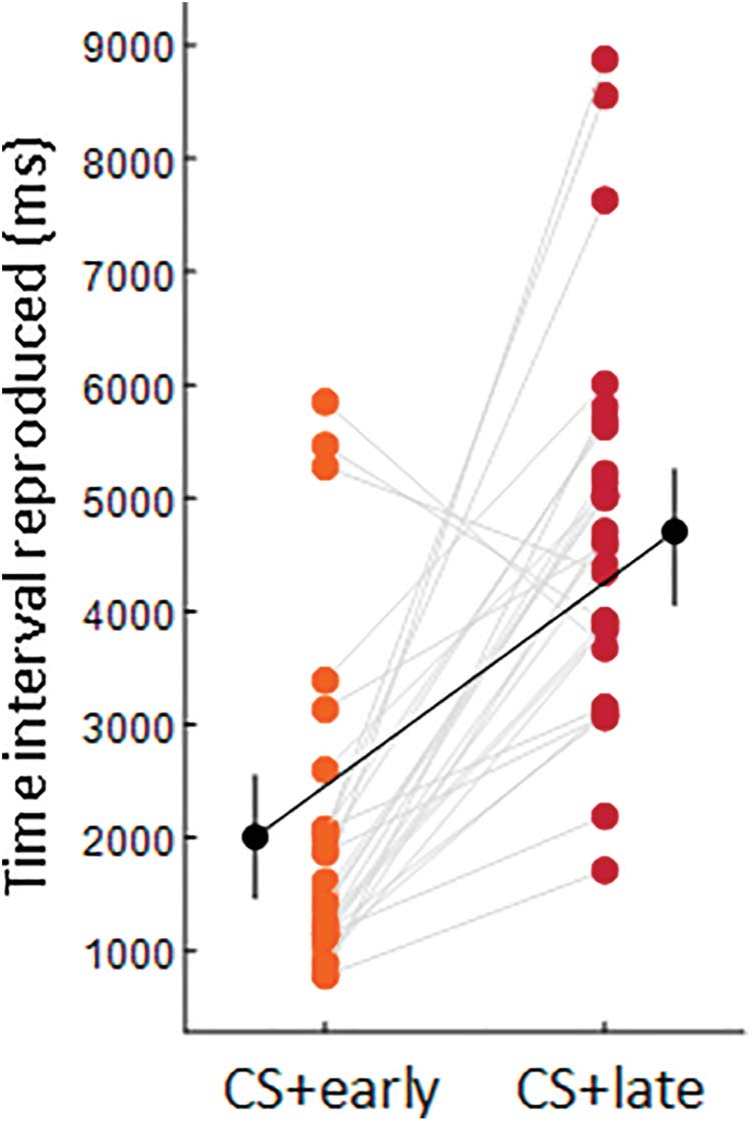
The plot shows individual participants' data (colored dots), group means (black dots), and 95% credible intervals (vertical black lines) of the time interval reproduced (ms) for CS+early (orange) and CS+late (red). Each paired set of observations is connected by a gray line.

A Bayesian paired sample *t* test comparing the mean time intervals reproduced for the CS+early and CS+late showed that participants reproduced a shorter interval for CS+early than CS+late (CS+early: M = 2006.707, 95% CI [1461.269-2552.146], CS+late: M = 4705.171, 95% CI [4055.134-5355.209]; BF_10_ = 38,394.492; err% = 2.089e-10), meaning that participants learned to discriminate the time of shock between the 2 CSs+ (Fig. 8). In addition, a Bayesian paired sample *t* test comparing the difference between the actual time of shock and the mean reproduced time interval for CS+early (1750 ms—mean reproduced time for CS+early) and CS+late (5500 ms—mean reproduced time for CS+late) revealed that this difference was smaller for CS+early than for CS+late (CS+early: M = −256.707, 95% CI [−802.146 to 288.731]; CS+late: M = 794.829, 95% CI [144.791-1444.866]; BF_10_ = 3.133, err% = 7.432e-7), indicating that participants were more accurate in reproducing the time interval for the early shock. Finally, a Bayesian paired sample *t* test comparing the standard deviation of time intervals reproduced for the CS+early and CS+late showed no difference in variability for the CS+early than CS+late (CS+early: M = 747.337, 95% CI [264.338-1230.335], CS+late: M = 1148.444, 95% CI [558.291-1738.596]; BF_10_ = 0.357, err% = 0.030); differently from experiment 1, participants exhibited similar variability in reproducing the time interval for the early and late shocks.

#### 3.2.3. Explicit acquisition of threat conditioning

Three Bayesian paired sample t-tests were performed comparing CS− and CS+ on valence, arousal, and shock contingency ratings, for both the Block early and Block late. After being conditioned to the CS+early, participants rated the CS+early as less pleasant (M = 2.607, 95% CI [1.98-3.235]) and more arousing (M = 7.929, 95% CI [7.381-8.476]) than the CS− (valence: M = 6.286, 95% CI [5.379-7.193]; BF_10_ = 26,179.312, err% = 4.886e-10; arousal: M = 1.536, 95% CI [0.864-2.207]; BF_10_ = 2.721e+11, err% = 1.279e-13). Regarding the CS−shock contingency, participants expected to receive the shock more in the presence of the CS+early (M = 7.036, 95% CI [6.582-7.489]) than the CS−early (M = 0.071, 95% CI [−0.075 to 0.218]; BF_10_ = 3.667e+19, err% = 3.767e-21). Similarly, after participants were conditioned to the CS+late, they rated the CS+late as less pleasant (M = 3.286, 95% CI [2.391-4.180]) and more arousing (M = 7.893, 95% CI [7.302-8.484]) than the CS− (valence: M = 6.429, 95% CI [5.537-7.321]; BF_10_ = 106.247, err% = 1.276e-4; arousal: M = 1.464, 95% CI [0.81-2.119]; BF_10_ = 6.972e+10, err% = 1.098e-13), and expected more the shock in presence of the CS+late (M = 7.286, 95% CI [6.869-7.692]) than the CS−late (M = 0.286, 95% CI [−0.23 to 0.801]); BF_10_ = 2.029e+16, err% = 3.745e-18).

### 3.3. Correlations combining data from experiments 1 and 2

Given the similarity of experiment 1 and 2 results, we then combined the data of the 2 experiments to explore, on a larger dataset, 2 sets of correlations. The first set tested the correlations between corticospinal excitability recorded at different timepoints during the CS+ presentation. The second set tested the correlations between corticospinal excitability and the time intervals elapsed between CS+ appearance and shock occurrence that were reproduced by participants.

Bayesian Pearson correlations were conducted to assess 2 sets of correlation, as described below. For all correlations, Cook distance (MATLAB package: influence.ME; function: influence; the output of the function influence was used in the function: cooks.distance) was used to estimate the impact of individual data points on the model outcome. Observations with Cook distance larger than 3 times the mean Cook distance were considered as outliers and excluded from the correlation.^81^ JASP 0.16^47^ software was used to obtain Bayes factors.

#### 3.3.1. Correlations between MEPs recorded at transcranial magnetic stimulation 1 and transcranial magnetic stimulation 2

**Figure 9.**
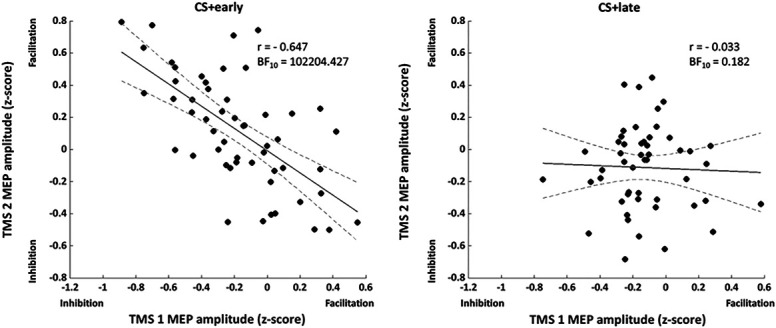
Correlations between MEPs recorded at TMS 1 and TMS 2. Dots represent single subject values, and dotted lines represent 95% confidence intervals.

These correlations tested the relation, across all participants, between the average motor evoked potential (MEP) amplitudes recorded at TMS 1 and TMS 2, separately for CS+early and CS+late. For CS+early, given the MEPs reduction at TMS 1 as compared to TMS 2 (Figs. 4 and 6), a negative correlation was expected. Cook distance analysis resulted in excluding 3 data points for the CS+early correlation (1 from experiment 1 and 2 from experiment 2) and 6 data points for the CS+late correlation (3 from experiment 1 and 3 from experiment 2). In Figure 9, the results are shown. A negative correlation for the CS+early (n = 53, r = −0.647, BF_10_ = 102,204.427) emerged, indicating that the lower the corticospinal excitability immediately before the time of shock, the higher the corticospinal excitability long after the time of shock. In contrast, no correlation emerged between corticospinal excitability probed at TMS 1 and TMS 2 for the CS+late (n = 50, r = −0.033, BF_10_ = 0.182).

#### 3.3.2. Correlations between MEPs and time reproduction

**Figure 10.**
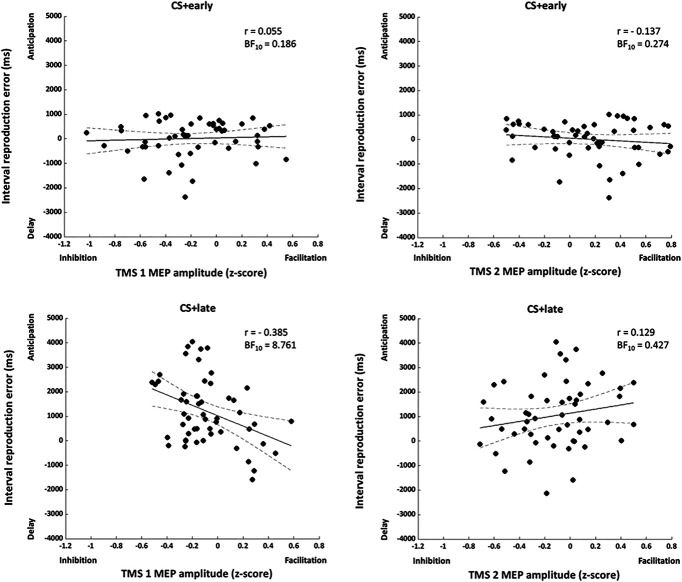
Correlations between MEPs and time interval reproduction error. Dots represent single subject values, and dotted lines represent 95% confidence intervals.

These correlations tested the relation, across all participants, between motor system excitability and participants' ability to accurately reproduce the interval between CS+ onset and shock occurrence. To this end, for each participant, an interval reproduction error was calculated by subtracting the time interval reproduced from the actual time of shock occurrence for the CS+early (1750 ms—reproduced time) and CS+late (5500 ms—reproduced time). Thus, a negative value indicated that participants delayed the time of shock, whereas a positive value indicated that participants anticipated the time of shock. The reproduction error was then correlated with MEPs amplitude at TMS 1 and TMS 2, for CS+early and CS+late. For CS+early, Cook distance analysis^81^ resulted in excluding 4 data points (1 from experiment 1 and 3 from experiment 2), both for the correlation with TMS 1 and TMS 2. No correlation was found between reproduction error and MEP at TMS 1 (n = 52, r = 0.055, BF_10_ = 0.186) or at TMS 2 (n = 52, r = −0.137, BF_10_ = 0.274, Fig. 10). For CS+late, Cook distance analysis^81^ resulted in excluding 3 data points (all from experiment 2) for the correlation that included MEPs at TMS 1 and 4 data points (1 from experiment 1 and 3 from experiment 2) for the correlation that included MEPS at TMS 2. A negative correlation was found between reproduction error and MEP at TMS 1 (n = 53, r = −0.385, BF_10_ = 8.761, Fig. 10). Thus, lower corticospinal excitability long before the time of the shock was associated with greater anticipation in participants' timing of the shock. No correlation was found between reproduction error and MEP at TMS 2 (n = 52, r = 0.129, BF_10_ = 0.427, Fig. 10).

## 4. Discussion

### 4.1. Experiment 1

Experiment 1 showed that the Pavlovian conditioning task was suitable to induce conditioning. Indeed, participants rated the CS+early and CS+late as less pleasant, more arousing, and more often associated with pain compared to the CS−. In addition, the time reproduction task showed that participants discriminated between the timing of pain associated with the CS+early and the CS+late.

Crucially, the results also confirmed that the sustained inhibition throughout pain anticipation hypothesis (H2) had the greatest support, indicating that the motor system encodes the interval between the onset of a threat and the time of pain occurrence.

### 4.2. Experiment 2

The results of experiment 2 conceptually replicated those of experiment 1, further confirming that changes in corticospinal excitability under the threat of pain reflect the timing of expected pain and that the motor system encodes the interval between the onset of a threat and the time of pain occurrence, demonstrating sustained inhibition during the pain anticipation period.

### 4.3. General discussion

In 2 experiments, we investigated whether the motor system learns the time of pain testing whether the inhibition of the motor system during pain anticipation^8^ is bound to the time of pain occurrence. To this end, corticospinal excitability was probed by administering spTMS over the left primary motor cortex, during a Pavlovian threat conditioning task in which the temporal imminence of pain^25,56^ was manipulated. Thus, as participants learned that initially neutral visual stimuli predicted pain early (CS+early) or late (CS+late) after their appearance, motor evoked potentials were elicited by spTMS at 3 critical timepoints during visual stimuli presentation. Two timepoints occurred during the pain anticipation interval, namely long and immediately before the time of pain, and one occurred long after the time of pain. We had 3 a priori hypotheses regarding the modulation of corticospinal excitability. The first 2 hypotheses posited that the motor system encodes the time of pain, but they differed in what aspect is encoded, namely, the precise time of pain or the interval between threat (CS+) onset and pain occurrence. In contrast, the third hypothesis posited that the motor system encodes the presence of a threat, but not pain timing. Bayesian informative hypothesis testing was used to test these hypotheses against each other and quantify how much more likely the data are under a given hypothesis as compared to the others.^28,37^ Finally, correlational analyses explored the relationship between the changes in corticospinal excitability under threat of pain, and the subjective estimates of the time elapsed between threat onset and pain occurrence.

We find that changes in corticospinal excitability acquired during threat conditioning are tied to the expected time of pain. Three key results support this conclusion. First, Bayesian hypothesis testing showed the strongest support for the hypothesis of sustained motor inhibition during pain anticipation. This dynamic of corticospinal excitability indicates that the motor system differentiates the period leading up to pain from the period afterward, encoding the interval between the onset of a threat and the expected time of pain occurrence through sustained inhibition. Second, corticospinal excitability appeared to recover long after the time of pain had passed, regardless of whether pain was actually administered. This time-bound recovery, even in trials without a painful shock, reinforces the interpretation that the motor system's response is linked to pain timing rather than the actual occurrence of pain. Third, correlations between corticospinal excitability and participants' subjective estimates of the interval elapsed from threat onset to pain occurrence revealed that stronger motor inhibition long before the time of pain was associated with greater distortion of pain timing, shifting subjective perception toward greater temporal anticipation of the actual time of pain.

In detail, during the pain anticipation interval, corticospinal excitability was reduced immediately before pain occurrence during CS+late presentation, replicating our previous finding^8^ showing that Pavlovian conditioning induces conditioned corticospinal inhibition measurable several seconds (>4000 ms) after CS+ onset. Here, we extend this finding, demonstrating that a similar reduction in corticospinal excitability also occurs immediately before pain in the CS+early condition, which has a much shorter pain anticipation interval (1690 ms). This result indicates that a brief anticipation interval is sufficient to reduce corticospinal excitability, suggesting that the learned threat value of a visual stimulus rapidly modulates motor system excitability. This aligns with the literature on intrinsic emotional stimuli,^10,71^ corroborating the tight coupling between visual processing and motor reactivity, particularly for stimuli that are motivationally relevant for survival.^45^ Critically, a reduction in corticospinal excitability was also observed long before the time of pain during CS+late presentation, suggesting early recruitment of the motor system during the pain anticipation period, already several seconds before pain is imminent. Importantly, replicating such a result in experiment 2 ruled out the possibility that the reduction in corticospinal excitability was because of a generalization effect of the pain-related temporal association established with the CS+early.^15,20,21,74^ Notably, a negative correlation emerged between corticospinal excitability long before the time of pain and participants' accuracy in reproducing the time of shock after the CS+late onset. Specifically, stronger motor inhibition long before the time of pain was associated with greater distortion of pain timing, with participants reproducing pain occurrence as having happened earlier than it actually did. Conversely, corticospinal facilitation was associated with a delayed reproduction of pain timing, suggesting that both inhibition and facilitation lead to opposite errors in time estimation. Overall, these findings indicate rapid recruitment of the motor system during pain anticipation, which occurs not only when the time of pain is imminent, but already long before that, soon after a threat of pain appears in the environment. In addition, they may suggest a role for the motor cortex in mediating the transformation of physical time into subjective time, when under threat.

Our results also showed the recovery of corticospinal excitability once the time of pain has passed, with such recovery being more pronounced when the inhibition during pain anticipation was stronger. These results further highlight the clear discrimination in the motor system of the intervals pre- and postpain, which aligns its excitability state with the expected time of pain. The recovery of motor excitability after the time of pain may be a marker of motor readiness for defensive action implementation. In addition, such recovery could represent a physiological marker of psychological relief, typically experienced when an expected negative event is omitted or terminated.^18^ Relief of aversive states, including pain, has previously been assessed by showing attenuation of startle^3,4,57,58,83^ and increase in skin conductance response.^86,87^ Here, we extend these findings by providing evidence of relief through changes in motor system excitability. Notably, our data suggest that this relief effect may be more pronounced after stronger anticipatory responses to pain.

The spatiotemporal proximity or urgency of expected pain critically determines which actions might be effective and how much time is available to choose among them.^25,56,77^ Temporal imminence thus plays a fundamental role in shaping the type, vigor, and timing of defensive responses.^39,62^ Our findings suggest that the motor system participates in pain anticipation across the temporal imminence continuum, being rapidly recruited when a threat for pain appears, until the expected time of pain occurrence. This is consistent with the role of the cortical motor system in event and action timing.^17,34,49,70^ Under threat of pain, the observed reduction in corticospinal excitability during pain anticipation may support the enactment of defensive responses. Anticipation of aversive events often triggers a threat-monitoring state marked by motor inhibition (eg, freezing), serving to enhance threat perception and action preparation^26,33,44,48^ and prevent temporally inappropriate reactions.^22–24,80^ Such an inhibitory state may be regulated by the prefrontal cortex,^50,60,61^ and removed at the expected time of pain, to enable the execution of a defensive response. Importantly, motor inhibition emerged seconds before pain and was related to distortions in pain timing that shifted toward its anticipation, suggesting a prediction system prioritizing preemptive inhibition over temporal precision. Notably, we observed effects in the FDI muscle in anticipation of shocks delivered on ECR, confirming that corticospinal inhibition can extend to functionally related muscles, likely as part of a preparatory or protective mechanism (in line with 8).

In conclusion, the cortical motor system encodes the time of pain modulating corticospinal excitability, by showing sustained motor inhibition throughout pain anticipation. Notably, stronger motor inhibition long before the time of pain was associated with greater anticipation of pain timing, whereas motor facilitation was associated with greater delay, suggesting this response may serve as a physiological marker for individual differences in pain timing predictions. Importantly, these modulations were not contingent on the immediate experience of pain but were driven by pain expectancy. Clinically, exaggerated motor inhibition or facilitation may undermine therapeutic efforts aimed at restoring motor function and managing pain, highlighting the importance of integrating motor system modulation strategies into pain management protocols. Future studies could further investigate the temporal dynamics of corticospinal excitability, disambiguating between the roles of central and spinal mechanisms in pain anticipation, and using alternative methodologies capable of capturing changes continuously rather than at discrete time points.

## Conflict of interest statement

The authors have no conflicts of interest to declare.

## Supplemental digital content

Supplemental digital content associated with this article can be found online at http://links.lww.com/PAIN/C331.

## Supplementary Material
