# Supplementary material for: Learning the time of pain in the human motor system
Source: Pain. 2025 Aug 6;166(12):e715–31. doi: 10.1097/j.pain.0000000000003730 (PMC12617657; doi:10.1097/j.pain.0000000000003730)
Supplement: Supplementary file 1 [file jop-166-e715-s001.pdf]

SUPPLEMENTARY MATERIALS

We conducted a preliminary experiment to test whether the experimental design used in experiments 1 and 2 was able to produce conditioning, measured through skin conductance responses [2]. Participants completed a Pavlovian threat conditioning task, during which they learned the association between three visual stimuli and their respective outcomes. The three visual stimuli were filled colored circles (64 pixels diameter, blue #5698D4, pink #C760CA, or yellow #F4E634), representing three different conditioned stimuli. Two of them were conditioned stimuli (CS+) associated with the delivery of an electrotactile shock to the right arm, representing the unconditioned stimulus (US), while the other stimulus (CS-) was never associated with shock. The procedure and statistical analysis follow what is described in Experiment 1. Unlike the main experiment, TMS pulses were not delivered to the participant's scalp, but the TMS noise was still presented and was produced using an intensity of 66% of the maximum stimulator output as the average value that emerged in a previous study [1]. This choice was intended to minimize differences with the experimental procedure adopted in the main study.

METHODS

Participants

Eleven healthy right-handed volunteers (7 women, aged between 18 and 30 years, M=24, SD=3.51 years) with the same characteristics as those participating in experiments 1 and 2 were tested.

Dependent variables

Skin conductance response (SCR). Galvanic skin conductance was recorded at 5000 Hz (gain switch set to 10, low-pass to 10 Hz), from pre-gelled snap electrodes (BIOPAC EL501) placed on the hypothenar eminence of the palmar surface of the left hand, connected to an EDA100C module of BIOPAC MP-150 System (Goleta, CA). The digitalized electrodermal activity signal was processed using Autonomate 2.8 (Green et al., 2014) running in MATLAB (The Mathworks) to obtain trough-to-peak SCR values. The digitalized signal was down-sampled at 625 Hz, an SCR was considered valid if the trough-to-peak response occurred between 500 to 5440 ms following the stimulus onset, lasted for a maximum of 5000 ms, and was greater than 0.02 µS. Raw SCR data were z-transformed, and values exceeded ± 2 SD of the sample mean were excluded as outliers. The raw SCR data were z-transformed separately for each participant to control for interindividual variability in SCR.

Time interval reproduced. The reproduced time intervals were recorded and preprocessed as in experiments 1 and 2.

Explicit acquisition of threat conditioning. Explicit ratings of threat conditioning were recorded and preprocessed as in experiments 1 and 2.

RESULTS

Skin conductance response (SCR). Only no-shock trials were included in the analysis to remove the impact that shocks administered during the CS+ presentation produced on SCR. The Bayesian rmANOVA was performed with CS (CS-, CS+early, CS+late) as a within-subject factor to analyze SCR data. A main effect of CS emerged (BF_{10}=97.691). Post-hoc comparisons highlighted that SCR was significantly higher for both CS+early (mean (M) = -0.219, 95% credible interval (CI) [-0.368, -0.069]) and CS+late (M=-0.317, 95% CI [-

0.418, -0.217]) than CS- (M=-0.513, 95% CI [-0.593, -0.432]; BF_{10}=12.008; err%=5.405e-6; BF_{10}=11.500; err%=4.941e-6, respectively), but did not differ between CSs+ (BF_{10}=0.660; err%=0.017; Figure 1).

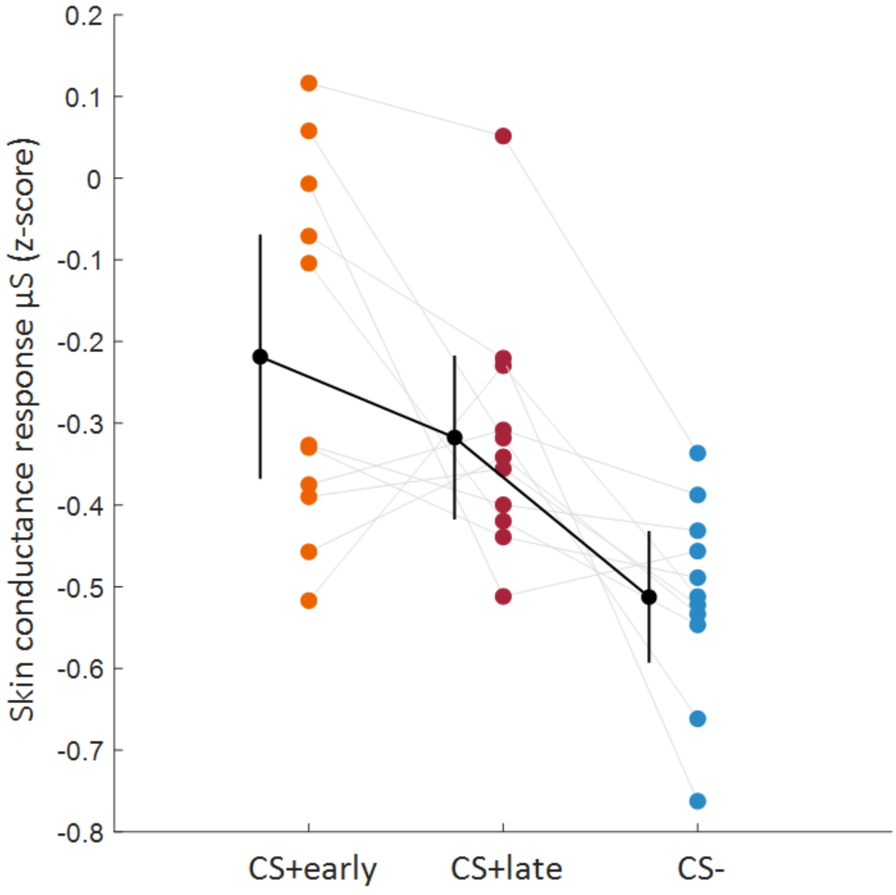

Figure 1. The plot shows individual participants' data (colored dots), group means (black dots), and 95 % credible intervals (vertical black lines) of SCR (µS) for CS+early, CS+late, and CS-. Each paired set of observations is connected by a gray line.

Time interval reproduced. A Bayesian paired t-test was performed to compare the mean of interval reproduced for the CS+early (M=2448.364, 95% CI [1274.488, 3622.239]) and CS+late (M=4148.218, 95% CI [2946.770, 5349.666]). We observed a difference in CSs (BF_{10}=20.558; err%=6.956e-7), meaning that participants learned the interval between the CSs+ onset and the shock delivery (Figure 2).

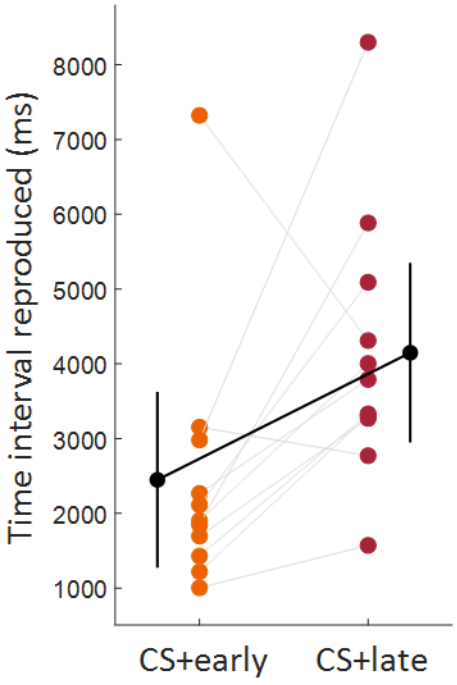

Figure 2. The plot shows individual participants' data (colored dots), group means (black dots), and 95 % credible intervals (vertical black lines) of the time interval reproduced (ms) for CS+early and CS+late. Each paired set of observations is connected by a gray line.

Explicit acquisition of threat conditioning. Three Bayesian rmANOVA and post-hoc comparisons were performed with CS (CS+early, CS+late, CS-) as a within-subject factor to analyze stimuli valence, shock expectancy and shock contingency ratings. Participants rated the CS- (M=7.455, 95% CI [6.203, 8.706]) as more pleasant than the CS+early and CS+late (BF_{10}=10096.182; err%=1.444e-8; BF_{10}=8425.968; err%=6.182e-8, respectively), whereas ratings for CS+early (M=2.36, 95% CI [1.269, 3.458]) and CS+late (M=1.45, 95% CI [0.487, 2.422]) did not differ (BF_{10}=0.685; err%=0.017). Moreover, participants rated as less arousing the CS- (M=0.818, 95% CI [0.093, 1.543]) compared to the CS+early and the CS+late (BF_{10}=5.162e+6; err%=1.230e-8; BF_{10}=1.013e+6; err%=9.375e-9, respectively), whereas ratings for CS+early (M=7.909, 95% CI [7.438, 8.380]) and CS+late (M=8.273, 95% CI [7.318, 9.227]) did not differ (BF_{10}=0.390; err%=0.009). Regarding the CS-US contingency, participants rated the contingency lower in the presence of the CS- (M=0, 95% CI [0, 0]) than the CS+early and CS+late (BF_{10}=1.444e+6; err%=4.915e-8; BF_{10}=266990.211; err%=2.604e-9, respectively), whereas ratings for CS+early (M=7.273, 95% CI [6.367, 8.179]) and CS+late (M=6.818, 95% CI [5.785, 7.851]) did not differ (BF_{10}=0.555; err%=0.014).

BIBLIOGRAPHY

[1] Betti S, Badioli M, Dalbagno D, Garofalo S, di Pellegrino G, Starita F. Topographically selective motor inhibition under threat of pain. Pain 2024;00:1–12.

[2] Lonsdorf TB, Menz MM, Andreatta M, Fullana MA, Golkar A, Haaker J, Heitland I, Hermann A, Kuhn M, Kruse O, Meir Drexler S, Meulders A, Nees F, Pittig A, Richter J, Römer S, Shiban Y, Schmitz A, Straube B, Vervliet B, Wendt J, Baas JMP, Merz CJ. Don’t fear ‘fear conditioning’: Methodological considerations for the design and analysis of studies on human fear acquisition, extinction, and return of fear. Neurosci Biobehav Rev 2017. Available: 10.1016/j.neubiorev.2017.02.026.
